# The value of adult orthodontics: Do the public’s willingness-to-pay values reflect the profession’s?

**DOI:** 10.1177/14653125211043124

**Published:** 2021-09-06

**Authors:** Kathryn Edwards, Jennifer Rae, Sarah Rolland, Christopher R. Vernazza

**Affiliations:** 1 Department of Orthodontics, Newcastle Dental Hospital, Newcastle University, Newcastle upon Tyne, UK; 2 Department of Orthodontics, Glasgow Dental Hospital, Glasgow, UK; 3 Department of Orthodontics, School of Dental Sciences, Newcastle University, Newcastle upon Tyne, UK; 4 Department of Paediatric Dentistry, School of Dental Sciences, Newcastle University, Newcastle upon Tyne, UK

**Keywords:** demographics, economics, resource allocation, patient preference, social values, Index of Orthodontic Treatment Need

## Abstract

**Objective:**

To explore how the public and dental professionals would value an orthodontic service for adults by eliciting their willingness-to-pay (WTP), a standardised health economics technique which quantifies ‘strength of preference’ in monetary terms. Despite increasing demand, adults in the UK are only eligible for NHS orthodontic treatment if there is severe dental health or complex multidisciplinary need. Orthodontic services are provided to children aged under 18 years who are eligible by their Index of Orthodontic Treatment Need (IOTN) score. Consequently, many adults who may have a need for treatment as determined by IOTN are unable to access this service.

**Design:**

Cross-sectional survey.

**Setting:**

General dental practices in North East England and national specialists approached through the British Orthodontic Society (BOS).

**Participants:**

Public participants were recruited from general dental practices. Dentists were recruited from local dental lists and members of the BOS.

**Methods:**

Participants were asked if they would be willing to pay to see an orthodontic service extended to all adults in England with a qualifying IOTN. Clinical photographs of three malocclusions were presented and maximum WTP in additional tax per household per year was elicited using shuffled payment cards.

**Results:**

A total of 205 dentists and 206 public participants were recruited. Pairwise tests showed a statistically significant difference in WTP between the public and professionals for all malocclusions, with the public giving higher valuations. In both groups, the Class III scenario elicited a higher WTP than the class I or II malocclusion. However, when all other factors were controlled for using a regression analysis, the group (public or profession) and the other variables did not significantly influence WTP.

**Conclusion:**

The public and professionals were willing to pay for an adult orthodontic service. Due to this variability and unpredictability the allocation of healthcare resources will remain contentious.

## Introduction

The demand for adult orthodontic treatment has increased in recent years (Pabari et al., 2011); however, there is little research investigating how adults value orthodontic care in quality of life or monetary measures (Smith and Cunningham, 2004), with none looking at how the dental profession would similarly value orthodontic care. Adults may seek orthodontic treatment for several reasons including missed opportunities as a child, orthodontic relapse, cosmetic reasons, indirectly as part of a multidisciplinary treatment plan, or to improve psychosocial wellbeing and self-esteem (Cedro et al., 2010). Currently, the provision of orthodontics in the taxation-funded National Health Service (NHS) in the UK is governed by the Index of Orthodontic Treatment Need (IOTN), to direct limited NHS resources to those with the highest perceived benefit. Adults are only eligible for treatment on a case-by-case basis if there is a severe dental health issue or complex multidisciplinary need (NHS England, 2015).

With increasing pressure on health service budgets, managers have difficult decisions to make regarding the provision of care. Even where adult orthodontics is unlikely to be wholly funded, decisions may need to be made as to whether to allow subsidised treatment or including in public or private insurance-based schemes. It is therefore important to quantify the level of benefit, or value, of treatments. There is minimal evidence to support that orthodontic treatment of some malocclusions provides a significant health gain (Benson et al., 2015); however, there is a perceived psychosocial benefit (Javidi et al., 2017) although this varies after treatment and does not relate to the extent of malocclusion corrected and is probably unpredictable in advance of treatment (Shaw et al., 2007). Although psychosocial benefit is important, benefit can also be defined more widely in terms of value, a measure of benefit commonly used in economics.

Willingness-to-pay (WTP) is a standard health economics technique that aims to quantify value in monetary terms (Cunningham and Hunt, 2000). One way of measuring WTP is using contingent valuation which measures the ‘strength of preference’ by asking the maximum that individuals would be prepared to pay for a certain form of health intervention via a hypothetical scenario, described by an interviewer or explained in a questionnaire (Gafni, 1991). When constructing a hypothetical WTP scenario, an ‘out-of-pocket’ payment vehicle is often used; however, this may limit participants to valuing only their individual health gain from the intervention depending on the nature of the intervention. Framing the question using increased taxation to fund a service allows for the valuation of external and societal health benefits (Birch et al., 1999; Srivastava et al., 2014). In orthodontics, this could be the benefit you ascribe to having the service available to your friends and family (or indeed, wider society), or peace of mind knowing the service is available should it be needed. When asking the general public to value the interventions being studied, they should provide more societally relevant results compared to patients or the profession. This is due to the familiarity and potential vested interest with the service that patients and the profession will have (Whitehead and Ali, 2010).

Most WTP studies in dentistry have focused on patient valuations for preventive therapy, or parents’ valuations regarding interventions relating to their children; some have also looked at community-based interventions (Tan et al., 2017). There is some research investigating how adults value orthodontic and combined orthognathic care (Cunningham and Hunt, 2000; Feu et al., 2012; Rosvall et al., 2009; Smith and Cunningham, 2004), though none have looked at how the dental profession would value orthodontic care. A systematic review of perceived need for orthodontics found a high variability in the treatment need perception among laypersons and specialist orthodontists and concluded that further studies are required to improve our understanding on perceived treatment need in orthodontics (Livas and Delli, 2013).

As the survey asked about increasing taxation to support orthodontic service provision, it would be reasonable to assume that the profession would have an implicit incentive to inflate their WTP values. Therefore, by comparing the public and professional values, this would provide insight into whether professional advocacy for increased services is in line with their own values or the values of the public.

The aim of the present survey-based study was to determine the values that the public and profession (general dentists and orthodontists) place on adult orthodontic treatment as a service by eliciting willingness to pay via increased taxation.

## Materials and methods

### Study design

A cross-sectional survey was designed to elicit WTP values from public participants across four general dental practices in North East England and professional participants (both general dentists and specialist orthodontists) from the North East and Cumbria and members of the British Orthodontic Society (BOS). Ethical approval was granted from the NHS North East Newcastle & North Tyneside Research Ethics Committee 2 (REC reference: 17/NE/0349).

### Survey design

The scenarios were developed in conjunction with hospital-based orthodontists at Newcastle University, Newcastle Dental Hospital and the Cumberland Infirmary, Carlisle to ensure content validity. Photos of patients with IOTN 4 malocclusions were chosen as they represent patients with a ‘great need’ for treatment and those who may benefit from orthodontic treatment. IOTN 5 was not chosen as they are more likely, in an adult population, to benefit from combined orthodontic and orthognathic treatment. The photos were chosen by the research team and reviewed by the Oral & Dental Patient and Public Involvement (PPI) Group from Newcastle University to ensure photos were representative of the malocclusions and outcomes. Investigation into orthognathic treatment was not the purpose of this study. Difficulty in understanding the hypothetical concept can affect WTP valuations, so the survey was piloted with members of PPI group to ensure comprehensibility and face validity of the survey.

All participants answered questions on demographics required for data analysis, based upon the Office for National Statistics (ONS) questions (Office for National Statistics, 2017). These were: age; gender; postcode (to determine index of multiple deprivation [IMD]); employment status; income; level of education; and ethnicity.

Professional participants were asked where they initially qualified, if they had any additional qualifications, and how long they had been qualified and practising. If they practised orthodontics, follow-up questions regarding the amount of adult and private orthodontics practised were asked.

The public sample completed the digital survey using Qualtrics software (Qualtrics, 2018) installed on university-provided tablet computers. The professional sample completed a web-based survey online via an anonymous email link. This carried a risk of repeat responses, but this risk was deemed to be very low.

### WTP elicitation

**Figure 1.**
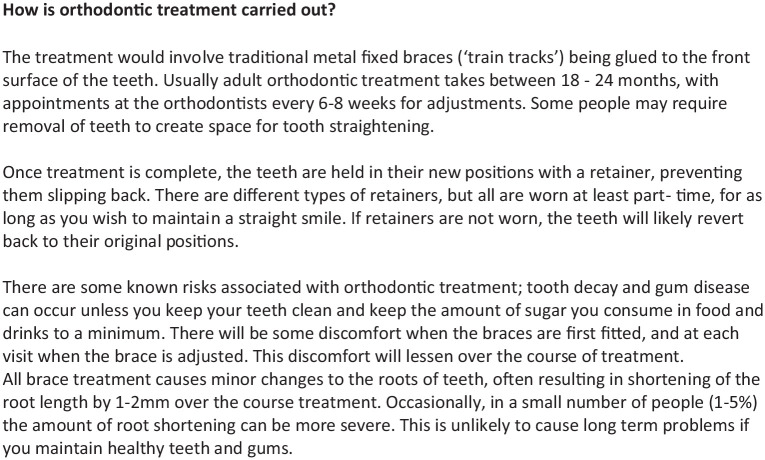
Wording used in the survey.

The WTP concept was explained at the start of the survey, clarifying that this was a theoretical exercise to investigate how adults value orthodontic treatment, not a tool to set a price or set tax levels (the full survey is available as supplementary material). In addition, what a typical course of orthodontic treatment involves and the associated risks and benefits were described (Figure 1) to ensure participants understood what orthodontic treatment encompasses.

**Figure 2.**
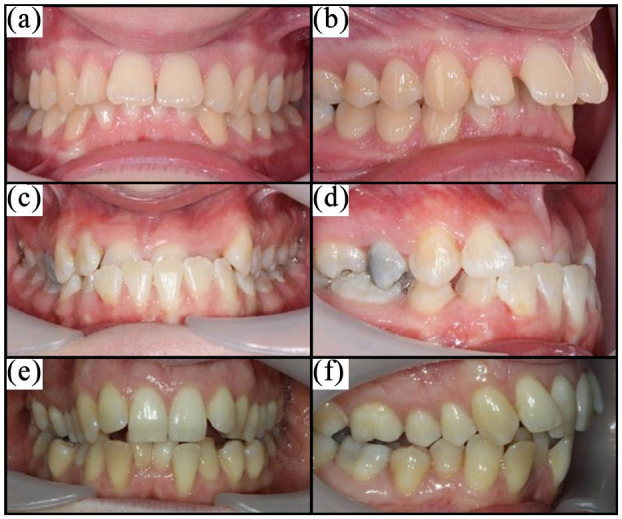
Photographs used for the scenarios: (a) IOTN 4d – Class I front view; (b) IOTN 4d – Class I side view; (c) IOTN 4c – Class III Scenario front view; (d) IOTN 4a – Class III Scenario side view; (e) IOTN 4a – Class II Scenario front view; and (f) IOTN 4c – Class II Scenario side view.

Standardised intra-oral clinical photographs (Figure 2) of three adults with Class I, II and III malocclusions (IOTN 4d, 4a and 4c, respectively) were presented to participants alongside a description of the nature of the service (fixed orthodontic treatment with metal brackets) and risks of treatment. Participants were asked if they would be willing to pay additional tax per household per year, to make available to all adults an NHS orthodontic service for correction of each malocclusion.

The script for the question encouraged realistic budget constrained responses. Maximum WTP in additional tax per household per year was elicited using a shuffled payment card method, with a range of £1–£200 (£1, £2.50, £5, £7.50, £10, £20, £30, £50, £100, £200) based on previous studies (Srivastava et al., 2014). Qualtrics software included the functionality of the shuffled method via a web-based questionnaire and was therefore the chosen software for the study.

If participants indicated they would be unwilling to pay any extra amount for the service, follow-up questions were asked to determine if the value was a true zero (i.e. the participant genuinely did not value the intervention) or a protest response (Arrow et al., 1993), where participants may have a value but are not willing to engage in the task (Ryan et al., 2004).

### Sample and recruitment

The sample size calculation was based on an Events Per Variable (EPV) approach for the logistic regression analysis (Peduzzi et al., 1996), where the requirement for a regression model is 10 participants per variable. Estimating 20 variables in each model, 200 public and 200 professional participants were therefore required.

The public participants were recruited consecutively between January and April 2018, from patients attending four general dental practices in North East England. The inclusion criteria included individuals aged 18 years or older who had capacity to consent for participation and were able to speak fluently in English. Adults unable or unwilling to consent where excluded. Participants could either complete the survey individually or face-to-face with a member of the research team. The professionals were recruited from the BOS list of members and from the Local Dental Committees list from the North East and Cumbria region. This was to recruit general dentists as well as specialist orthodontists. They completed the survey online and participants could email if they had any questions. Survey completion took approximately 20 min and participants were free to withdraw from the survey at any time. No financial incentives were offered, minimising recruitment bias.

### Statistical analysis

Data were automatically collected using the digital survey; it was then imported into STATA software (StataCorp, 2017) for analysis. The minimum level for statistical significance was set at *P* < 0.05.

WTP for each IOTN scenario was analysed descriptively, comparatively and econometrically. Both parametric and non-parametric measures are presented in line with standard practice for WTP data (Carson et al., 2001). A Wilcoxon rank sum test was used to determine the significance of difference in WTP between the different groups (public and profession) and to investigate binary demographic data. To compare within groups (between the Class I, II and III scenarios), a Wilcoxon matched pairs signed-rank test was used.

A regression analysis was carried out to analyse the variables predicting WTP values while controlling for the others, accounting for confounders. The participant’s postcode was used to calculate their IMD decile using an online tool (Ministry of Housing Communities and Local Government, 2015). Missing postcodes were excluded from the analysis. Employment status was determined based on the National Statistics Socioeconomic Classification (NS-SEC) (Office for National Statistics, 2017). Dummy coding was used for multilevel categorical variables (IMD, income, qualification, professional qualifications, years qualified, place qualified and workload) to provide binary variables. Tobit regression models were used due to censoring of values at zero (i.e. it is impossible to have a negative WTP and consequently data are positively skewed) and a backwards stepwise elimination (i.e. starting with all variables of interest and eliminating these one by one until the best fitting model is found) comparing pseudo r^2^ between models was used to select the best fitting model.

## Results

A total of 206 public participants were recruited into this study. Across the public sample there was good representation of different groups with the group being similar to the local population (Table 1). The mean age for the public participants was 45 ± 16 years (age range = 18–84 years).

**Table 1.** Demographics of the sample.

	Public participants	Professional participants	ONS average (%) (ONS, 2011)
*Gender*			
Male	74 (36)	54 (26)	49
Female	132 (64)	59 (29)	50
Missing	0 (0)	92 (45)	
*IMD*			
IMD 1–4 (low IMD/ most deprived)	103 (50)	12 (6)	Data unavailable
IMD 5–7 (medium IMD)	38 (18)	23 (11)	Data unavailable
IMD 8–10 (high IMD/ least deprived)	58 (28)	33 (16)	Data unavailable
Missing	7 (3)	137 (67)	
*Income*			
High income (£31,200+)	54 (26)	105 (51)	Data unavailable
Medium income (£15,600– £31,199)	70 (34)	4 (2)	Data unavailable
Low income (£0–£15,599)	80 (39)	4 (2)	Data unavailable
Missing	2 (1)	92 (45)	
*Qualification*			
Degree	51 (25)	193 (94)	27
Diploma + A levels	75 (36)	0 (0)	12
GCSE at C	48 (23)	0 (0)	15
Other/none	21 (10)	0 (0)	18
Missing	11 (5)	12 (6)	

Values are given as n (%) unless otherwise indicated.

IMD, index of multiple deprivation; ONS, Office for National Statistics.

In total, 205 professional participants were recruited to the study. Of these, 101 (49%) had incomplete surveys, mainly relating to non-completion of demographic questions, but available data from these responses were used. Of them, 173 (84%) said they practised orthodontics but it was not clear at what level. The mean age of the professional participants was 44 ± 11 years (age range = 26–71 years; 92 had missing data for age).

Mean and median WTP values for each of the scenarios are shown in Table 2, along with the number of protest responses and true zeros. Median WTP values were used as the data were non-parametric. Mean WTP valuations were also appropriate for comparison as WTP data can be considered continuous in nature and econometric modelling utilises mean valuations (Carson et al., 2001). Mean values were considerably higher than the median values, demonstrating skew in the data. Protest responses were excluded from analysis to minimise biasing of the results. The results show the public and profession were willing to pay for an adult orthodontic service on the NHS through increased taxation.

**Table 2.** Summary of descriptive statistics for WTP for each scenario.

	Value given	Protest zero	True zero	WTP (£)	WTP (£)
*Public sample (n = 206)*					
Class I	168	34	4	57.6 ± 70.8	30 (90.0)
Class II	164	36	6	59.0 ± 74.7	30 (90.0)
Class III	169	33	4	68.7 ± 82.1	50 (85.0)
*Profession sample (n = 205)*					
Class I	75	111	19	35.2 ± 91.8	8 (19.0)
Class II	73	118	14	40.0 ± 87.8	10 (27.5)
Class III	89	112	4	55.7 ± 95.1	20 (42.5)
*Combined sample (n = 411)*					
Class I	243	145	23	49.7 ± 79.4	20 (42.5)
Class II	237	154	20	52.6 ± 79.7	30 (42.5)
Class III	258	145	8	64.2 ± 86.9	30 (90.0)

Values are given as n, mean ± SD or median (range).

WTP, willingness-to-pay.

A Wilcoxon rank sum test was used to determine whether there was a difference in mean WTP values between the public and profession for each of the scenarios. There was a statistically significant difference in WTP between the public and the profession (*P* < 0.0001 for Class I and Class II scenarios, *P* = 0.0083 for Class III), with the public providing a higher value.

To determine if participants’ mean WTP values were influenced by the malocclusion, a Wilcoxon matched pairs signed-rank test was used to compare the different scenarios within the groups. In the professional sample, there was a significant difference between mean WTP values for Class I and Class II (*P* = 0.0339), Class I and Class III (*P* < 0.0001), and Class II and Class III (*P* < 0.0001). In the public sample, there was a statistically significant difference between Class I and Class III (*P* < 0.0001) and Class II and Class III (*P* < 0.0001). In all cases, the Class III scenario elicited the highest value, and the Class I scenario the lowest value.

To investigate the demographic or professional factors that may influence WTP, regression analyses were carried out for each malocclusion and each group as well as for the combined overall sample, an example of which (Class III) is shown in Table 3 (independent variables explained in Table 4). In the professional sample regression, all models overall showed a poor fit, evidenced by low pseudo R^2^ values, meaning that most of the difference is not explained by the variables. In all scenarios, those practising orthodontics for 10–20 years gave significantly higher WTP values. In the Class III scenario, the only significant variable was having a qualification at A level with those having A level or above having lower WTP value (Table 3).

**Table 3.** Tobit regression model for combined sample for WTP for the Class III scenario (number of observations = 218, pseudo R^2^ = 0.0059).

Class III	Coefficient	Standard error	T	p>t	95% confidence interval	
					Lower bound	Upper bound
Profession	−4.29	20.22	−0.21	0.832	−44.16	35.58
18–30	−9.02	17.88	−0.50	0.614	−44.27	26.23
31–40	−9.22	18.62	−0.50	0.621	−45.93	27.49
41–50	20.56	17.09	1.20	0.231	−13.14	54.25
Degree	−9.71	24.71	−0.39	0.695	−58.43	39.01
A Level	−46.42	22.53	−2.06	0.041	−90.85	−2.00
GCSE	−13.09	24.10	−0.54	0.588	−60.61	34.43
High income	−16.15	18.78	−0.86	0.391	−53.17	20.87
Med income	−6.99	18.62	−0.38	0.708	−43.69	29.71
High IMD	−24.94	15.97	−1.56	0.120	−56.44	6.55
Med IMD	−22.99	16.61	−1.38	0.168	−55.74	9.76
Male	−0.77	13.64	−0.06	0.955	−27.66	26.12
Constant	112.38	24.70	4.55	0.000	63.69	161.08
Sigma	8267.69	803.72	–	–	6825.69	10014.32

IMD, index of multiple deprivation; WTP, willingness-to-pay.

**Table 4.** Description of profession dummy variables for regression analysis.

Descriptor	Comparator	Number in group
FDS	Compared to MOrth, Postgraduate or no orthodontic qualification	54
Morth	Compared to FDS, Postgraduate or no orthodontic qualification	82
PGrad	Compared to FDS, MOrth or no orthodontic Qualification	22
Ortho 20+	Compared to practising orthodontics for 10–20 years and <10 years	64
Ortho 10-20	Compared to practising orthodontics for 20+ years and <10 years	50
Private Ortho	Practising a substantial amount of private orthodontics (25%–100% of orthodontic workload) compared to practising some private orthodontics (0%–25% of orthodontic workload)	52
Adult Ortho	Practising a substantial amount of adult orthodontics (25%–100% of orthodontic workload) compared to practising some adult orthodontics (0%–25% of orthodontic workload)	77
UK	Primary dental qualification obtained in the UK compared to primary dental qualification gained outside the UK	77

## Discussion

This study showed that there was a significant difference between the public and profession WTP for an adult orthodontic service, with the public giving a higher value in a simple two-way analysis. This contrasts with the values expected from the profession who it could be assumed would have professional incentives to inflate the value of treatment. However, this difference was lost when all other factors, including confounding factors, were controlled for in the regression analysis. The regression models all showed a poor fit; therefore, most of the difference in WTP was not explained using the variables collected. Future qualitative research may be useful to elicit which factors could be considered important.

Although WTP has been suggested as an appropriate preference-based measure in dentistry (Birch and Ismail, 2002), there are several criticisms associated with this approach, both practical and methodological. The WTP response depends on accuracy, completeness and clarity of the information provided to participants to ensure valid and realistic WTP values are obtained (Birch and Ismail, 2002). A pre-tested script was employed, detailed description of the intervention given, and the study piloted before finalisation. Some minor changes to the final wording were required, helping ensure respondents give their true WTP and not just guess the cost of the intervention. To illustrate what orthodontic treatment can achieve, only one intra-oral illustrative clinical photograph of an ‘ideal outcome’ was presented. However, this ‘ideal’ outcome may not be achievable in all adults due to complicating factors such as restored or missing teeth, tooth wear, slow tooth movement, periodontal problems and a lack of growth (Christensen and Luther, 2015). The values given therefore are for the ‘ideal’ outcome only.

Extra-oral photographs were not included in this study to allow for standardised cropping of the photos for each scenario, and to help participants focus on the severity of malocclusion rather than facial characteristics. This may also prevent potential misconceptions regarding facial aesthetic changes associated with orthodontic treatment alone, which has been shown to influence WTP (Smith and Cunningham, 2004).

The photographs illustrated the aesthetic benefits of orthodontic treatment, but as the functional and psychological benefits are difficult to quantify for this type of survey and were not explained, some patients may be less aware and would be basing their valuations on appearance alone. One possible concern with the valuations given is that the long-term outcomes are also difficult to quantify and be certain about. It is possible that respondents will have assumed that the final result was a permanent feature, which may or may not be true. Professional participants may be more likely to be aware of the functional and psychological implications of each of the malocclusions. This is suggested by the low mean WTP for the Class I scenario as compared to the Class II or Class III scenario in the professional group.

The professional participants may base their values on their own experience of carrying out orthodontics, as there was a large proportion of respondents who practised orthodontics. This would be a mixture of specialists and non-specialists, as we did not capture data on specialist status, other than in ‘qualifications’.

Several methodological approaches were employed to minimise bias inherent to WTP studies. Shuffled payment cards reduce starting point and range bias, and a taxation payment vehicle reduces anchoring of values on actual prices (Kahneman et al., 1999). This was of importance here as adult orthodontics is often privately funded and patients with this knowledge may have focused on the cost of treatment rather than their own valuation of treatment. The relatively low mean and median values given, and lack of very high values, suggests that participants understood the concept of the taxation-based question. However, the issue of scope, a common problem in WTP studies (Carson and Mitchell, 1993) remains, with a single service being valued in isolation. Individuals were asked to consider their WTP within their own budgetary limitation, but the exercise does not ask participants to consider their preferences for allocating their spend to other services. This must be borne in mind when interpreting the results.

In this study, some of the valuations were substantially higher than expected but hypothetical scenarios are necessary where a perfect market does not exist to reveal preferences (Donaldson, 2001). WTP values may also be susceptible to anchoring bias if participants have prior knowledge of prices related to the hypothetical scenario, again influencing validity. This was less likely to be the case in this study with a taxation-based payment vehicle.

WTP is associated with ability to pay and this can lead to skewed results if the sample is not representative. Although the professional sample had increased levels of high-income participants, they had a lower WTP, and no significant differences in WTP were seen across the income or IMD levels when the public and professional sample was analysed together in the regression analysis, suggesting this effect was minimal. Public participants were recruited from general dental practices in the North of England and may not be considered a representative sample of the population. The research team visited on weekdays during normal office hours (09:00–17:00), which may have limited the working population captured (Donaldson, 2001). This convenience sampling method may have introduced a selection bias and restricting the recruitment to one geographical area may also reduce the generalisability of the results.

In addition, the recruitment strategy meant responders were more likely to be regular dental attenders and may have increased knowledge of orthodontic treatment, which may represent the individuals most likely to be referred to the orthodontist.

To gain an insight into participants’ decision-making process and explore the methodological biases associated with the WTP technique, future qualitative research in combination with a WTP survey would facilitate the interpretation and validation of quantitative results. A larger sample may provide a better representation of the general population and improve the external validity and generalisability of these results. This information could then be utilised in a cost–benefit analysis to establish whether this proposed service has a potential place in the NHS, even with a patient co-payment, or another health system including private insurance-based systems and aid policy makers in evolving and designing healthcare systems that meet the public’s needs.

Measuring and valuing the costs and benefits of healthcare interventions has become increasingly important to ensure efficient use of limited healthcare resources (Cunningham, 2000). Little research has been done using economic preference measures in orthodontics despite being an area where its value in health systems has been controversial. Dental professionals may place different values on certain malocclusions compared to the general public (Rayner et al., 2015) and so health policy makers should take into account the opinion of both the profession and public when determining healthcare allocation (Livas and Delli, 2013). However, with the large variance seen in this study and the unpredictability by demographic factors, it is difficult for policy makers to make decisions on resource allocation across the whole population regarding provision of adult orthodontics.

## Conclusion

The public and profession were willing to pay for an adult orthodontic service. Correction of a Class III malocclusion was valued significantly more than correction of a Class I or II malocclusion at moderate levels of need; however, there was considerable variability in the data. There was considerable variance in the values that was not predicted by demographic or professional demographic variables. This information will be beneficial in the prioritisation of resources to fund patient-centred orthodontic treatment. However, large variance and unpredictability makes it difficult from a resource allocation perspective.
